# Agreement between triage category and patient’s perception of priority in emergency departments

**DOI:** 10.1186/s13049-016-0316-2

**Published:** 2016-10-18

**Authors:** Ghasem-Sam Toloo, Peter Aitken, Julia Crilly, Gerry FitzGerald

**Affiliations:** 1 School of Public Health and Social Work, Queensland University of Technology, Victoria Park Road, Kelvin Grove, QLD 4059 Australia; 2 Townsville Hospital Emergency Department, James Cook University, 1 James Cook Drive, Townsville, QLD 4811 Australia; 3 Department of Emergency Medicine, Menzies Health Institute, Queensland, Griffith University, Gold Coast Hospital and Health Service, 1 Hospital Boulevard, Southport, QLD 4215 Australia

**Keywords:** Emergency department, Inter-rater agreement, Triage, Patient perception, Perceived priority

## Abstract

**Background:**

Patients attending hospital emergency departments (ED) commonly cite the urgency and severity of their condition as the main reason for choosing the ED. However, the patients’ perception of urgency and severity may be different to the nurses’ perception of their urgency and severity, which is underpinned by their professional experience, knowledge, training and skills. This discordance may be a cause of patient dissatisfaction. The purpose of this study is to understand the extent of agreement/disagreement between the patient’s perceived priority and actual triage category and associated factors.

**Methods:**

A cross-sectional survey of 417 patients attending eight public hospital EDs in Queensland, Australia between March and May 2011 was conducted. The survey included patient’s perceived priority and other health-related, socio-demographic and perceptual factors. Patients’ triage category data were retrieved from their ED records and linked back to their survey data. Descriptive and multinomial logistic regression analyses were used.

**Results:**

Over 48 % of the respondents expected to be given higher priority than the actual triage category they were assigned; 31 % had their perceived priority matched with the triage category; and 20 % of the respondents expected a lower priority than the triage category they received (Kappa 0.07, *p* < 0.01). Patients who expected a higher priority tended to be more frequent users (≥3 times in the past six months), and to score higher on perceived seriousness, perceived urgency, and pain score compared to the patients whose perceived priority matched the triage category or anticipated a lower priority. In the multivariate analysis, only perceived urgency remained significantly associated with expected higher priority (OR = 1.27, 95 % CI: 1.14–1.43).

**Discussion:**

Our findings clearly confirmed the discrepancy between patient perception of urgency and staff assessment of urgency. This can have important implications particularly for the patients who underrate the urgency of their condition. Improved and open communication and the incorporation of the ‘patient voice’ into the triage process require understanding the patient’s perspectives and their involvement in the decision making process.

**Conclusions:**

Noted differences between patient and practitioner perception of clinical urgency were identifed in this study.

## Background

While hospital emergency departments’ (EDs) primary role is to treat the acutely ill and injured, they also provide a safety net for people who do not have access or cannot afford private health services [1, 2]. This coincides with the rising demand for and use of EDs in many countries including Australia [3, 4]. Although many factors contribute to this increase, some literature point to “inappropriate” users [5–9] who are also referred to as “primary care” or “general practitioner (GP) type” patients. Advocates of this opinion use criteria such as low triage category and lack of the need for admission into the hospital wards to assert that these patients do not need to be in an ED in the first place and should visit a primary health care service instead [10, 11]. On the other hand, opponents of this view assert that the claims of inappropriateness based on *post hoc* diagnoses are themselves inappropriate as patients are neither equipped with medical knowledge and skills nor should they be expected to be able to self-diagnose and identify the type of medical care that suits them [10, 11].

An ED or emergency care centre is defined as a place where people who perceive a need for urgent health advice or treatment turn to [12, 13]. On arrival to an ED, patients should be seen and briefly assessed by a triage nurse who assigns them a triage category 1–5 based on the Australasian Triage Scale (ATS) indicating how urgently the patient is to be attended by the medical staff (ATS-1 = immediately; ATS-5 = within 2 h) [14]. With studies showing that patient satisfaction is positively related to better access to the health system [13], and good communication with the staff [15–17], a discrepancy between the patient’s and practitioner’s views at the point of triage can become an early source of dissatisfaction and complaints for a patient and their family. An Australian study undertaken 10 years ago found that while the participants did not have a clear understanding of how the triage system worked, they wanted to know their initial triage category and receive ongoing information about it [18]. In today’s patient-centred approach to health care delivery, further research is required in this area.

Patients attend EDs because they see their condition as urgent and requiring medical attention [19–23]. However, the triage nurse or doctor who examines the patient may not share the patient’s sense of urgency. Furthermore, doctors and nurses can disagree over a patient’s triage category [18, 24–26]. Thus, expecting the patient to share a similar view with the triage nurse or doctor and agree with their decision can prove difficult. This suggests that there may be a discrepancy between patient’s perception and nurse’s perception of acuity; however further work is required to more definitively understand this.

The aim of this study is to assess the degree of agreement between patients’ perceived priority and actual triage category in the ED. Factors that may explain any concordance or discordance will also be explored.

## Methods

**Fig. 1:**
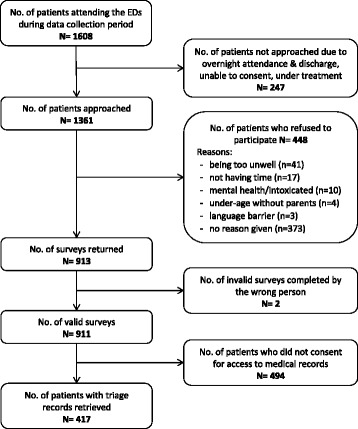
Flow chart of data collection process

Detailed information about the study design, population, sampling, data collection, theoretical framework and measures have been previously published [23, 27, 28]. In summary, a cross-sectional survey of ED patients was conducted in eight public hospitals across Queensland, Australia. Four of the hospitals were located in major cities, two in inner regional areas, and two were in outer regional and remote areas. One ED was a children’s ED and all others treated both adults and children. The data collection was conducted by four members of the research team and a group of eight trained interviewers in March to May 2011 (corresponding to autumn season). Data collection took place between 8 am and 10 pm on at least two midweek and one weekend days in each ED to capture a variety of patients. Of the total of 1608 patients in all eight emergency departments the interviewers were able to approach 1361 patients (85 %) and seek their consent to participate in the study. In total, 911 valid surveys were collected for the study, out of which 417 consented for their medical records to be accessed for this analysis (response rate = 31 % of those approached). Figure 1 shows the data collection process.

As part of the self-assessment questions, the respondents were asked what priority they thought they should be given based on their perceived severity and urgency of their condition. The response options were aligned with the recommended Time-to-be-Seen by the Australasian Triage Scale, that is: (1) Immediately; (2) Within 10 min; (3) Within 30 min; (4) Within 60 min; and (5) Within 2 h [14].

To be able to compare patient responses with their actual triage category, participants were requested to provide consent for their medical records to be accessed by the research team. The triage category for the patients who consented was retrieved from the Emergency Department Information System (EDIS), and added to their survey responses.

All data were managed and analysed using IBM SPSS Statistics V21.0 (IBM Corp.). Univariate analysis, Crosstabs, Chi-squared and F tests were used to analyse the data and bivariate associations of the dependent variable (i.e. agreement between patient perceived priority and actual nurse assigned triage category) with independent factors. Kappa, Tau-b and Gamma statistics were used to assess the strength of the inter-rater agreement. All factors that had a statistically significant association with the dependent variable at p ≤0.05 were entered into a Multinomial Logistic Regression to estimate the relative strength of predictor variables, using the Main Effects model with Forward Entry, and Confidence Interval (CI) set at 95 %.

## Results

A total of 911 valid surveys were collected, of whom 45.8 % (*n* = 417) consented for their triage category to be retrieved from their medical records. As Table 1 shows, the consented and non-consented respondents were not significantly different in terms of their general demographic and perceived health characteristics suggesting representativeness of the sample and the ability to generalise the findings to the broader patient population.

**Table 1:** Respondents’ characteristics by consent group

Characteristic	Consented		Non-consented		*χ* ^2^ (p)
	*n*	*%*	*n*	*%*	
Respondent					
- Patient	309	45.0	378	55.0	0.71 (0.22)
- Parent/Carer	108	48.2	116	51.8	
Age (year)					
- Mean	42.2		43.9		F test: 1.79
- Standard Deviation	18.3		18.4		(0.18)
Gender					
- Male	188	46.8	214	53.2	0.20 (0.35)
- Female	224	48.3	240	51.7	
Indigenous status					
- Indigenous^a^	23	54.8	19	45.2	0.66 (0.26)
- Non-Indigenous	389	48.3	416	51.7	
Country of birth					
- Australia	322	49.2	333	50.8	2.36 (0.07)
- Other	90	43.1	119	56.9	
Household weekly income					
- $1–249	23	54.8	19	45.2	2.46 (0.65)
- $250–599	87	51.5	82	48.5	
- $600–999	104	52.0	96	48.0	
- $1000–1599	84	45.7	100	54.3	
- $1600,+	65	52.4	59	47.6	
Highest education					
- None/Primary	46	54.1	39	45.9	2.63 (0.27)
- High school/ Trade	207	46.7	236	53.3	
- Tertiary	157	51.6	147	48.4	
General health status					
- Poor	22	45.8	26	54.2	1.25 (0.87)
- Fair	55	50.9	53	49.1	
- Good	113	46.3	131	53.7	
- Very good	122	44.9	150	55.1	
- Excellent	100	45.2	121	54.8	
Patient perceived priority					
1 - Immediately	54	49.5	55	50.5	3.80 (0.43)
2 - Within 10 min	63	42.9	84	57.1	
3 - Within 30 min	128	44.1	162	55.9	
4 - Within 60 min	100	50.3	99	49.7	
5 - Within 2 h	61	50.8	59	49.2	
ED use in past 6 months					
- None	228	46.1	267	53.9	1.80 (0.41)
- 1–2 times	132	48.9	138	51.1	
- 3,+ times	53	41.7	74	58.3	
Total^b^	417	45.8	494	54.2	

^a^Includes Aboriginals and Torres Strait Islanders

^b^Where the sum of sub-categories does not equal the Total, indicates missing values

As Table 2 shows, in 30.9 % of the respondents’ who consented to medical record access, perceived priority matched the triage category (bold italic cells); 48.5 % of expected to be given higher priority (italic cells) than the actual triage category they were assigned; and 20.6 % of the respondents expected a lower priority than the actual triage category. Although there is a tendency for patients to over-rate their priority, the correlation between perceived priority and actual triage category is weak (based on Tau-b and Gamma), and the Kappa shows no statistically significant agreement between the two variables.

**Table 2:**
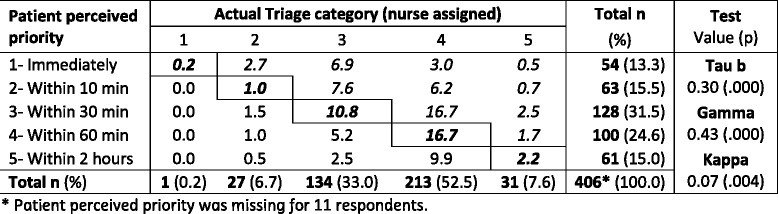
Agreement between perceived priority and triage category (% of Total)

### Factors associated with Inter-rater agreement

The concordance between the two variables (i.e. Patient’s perceived priority and Actual triage category) was also considered in three categories for further analysis:

1. Expected higher priority, includes those who received a lower triage category than their perceived priority (*n* = 197);
2. Concordance, includes those whose triage category matched their perceived priority (*n* = 126);
3. Expected lower priority, includes those who received a higher triage category than their perceived priority (*n* = 83).

We tested the bivariate associations between inter-rater agreement and other variables including socio-demographic factors (age, gender, employment status, income, education, country of birth, Indigenous status, living arrangement, social support, self-efficacy), health related factors (perceived health status, urgency, seriousness and pain, previous ED attendance, arrival method) and reasons for attending ED. Table 3 shows the factors that were significantly associated with inter-rater agreement (only statistically significant associations with *p* ≤ 0.05 have been displayed).

**Table 3:** Inter-rater agreement and associated factors

	Inter-rater agreement			
Factor	Expected higher priority	Concordance	Expected lower priority	Test (p)
ED use in past 6 months	% (95 % CI)	% (95 % CI)	% (95 % CI)	
- None	42.2 (35.9–48.7)	35.9 (29.9–42.4)	22.0 (17.0–27.8)	*χ*2 = 11.63
- 1–2 times	53.4 (44.9–61.8)	24.4 (17.9–32.4)	22.1 (15.9–30.0)	(0.02)
- 3,+ times	62.7 (49.0–74.7)	27.5 (17.1–40.9)	9.8 (4.3–21.0)	
Needed urgent care				
- Not considered	34.0 (22.2–48.3)	42.6 (29.5–56.7)	23.4 (13.6–37.2)	*χ*2 = 16.51
- Considered to some extent	40.4 (32.7–48.7)	32.6 (25.4–40.7)	27.0 (20.3–34.8)	(0.002)
- Considered to a great extent	58.0 (51.2–64.6)	25.9 (20.3–32.2)	16.1 (11.7–21.7)	
Perceived seriousness (1–10)				
- Mean (95 % CI)	7.1 (6.8–7.4)	6.0 (5.6–6.3)	6.1 (5.7–6.5)	F = 14.23
- Standard Deviation	2.1	2.0	1.9	(<0.001)
Perceived urgency (1–10)				
- Mean (95 % CI)	7.4 (7.1–7.7)	6.2 (5.8–6.6)	6.0 (5.5–6.5)	*F* = 17.11
- Standard Deviation	2.2	2.2	2.3	(<0.001)
Pain score (1–10)				
- Mean (95 % CI)	6.5 (6.2–6.9)	5.7 (5.2–6.2)	5.3 (4.7–5.9)	*F* = 7.65
- Standard Deviation	2.6	2.8	2.6	(<0.001)
Total (%)	197 (48.5)	126 (31.0)	83 (20.4)	406^a^ (100.0)

^a^Patient perceived priority was missing for 11 respondents

Expected higher priority was significantly associated with less frequent ED attendance in the past six months (*χ*2 = 11.6, *p* = 0.02) and stronger perceived need for urgent care (*χ*2 = 16.5, *p* = 0.002). Also, respondents who expected a higher priority than the assigned triage category were significantly more likely to perceive their condition as serious (mean score = 7.1 ± 2.1, *p* ≤ 0.01), urgent (mean = 7.4 ± 2.2, *p* ≤ 0.01), and painful (mean = 6.5 ± 2.6, *p* = <0.01) than the other two categories.

Expected lower priority was significantly associated with frequent ED attendance in the past six months (*χ*2 = 11.6, *p* = 0.02) and weaker perceived need for urgent care (*χ*2 = 16.5, *p* = 0.002). Also, respondents who expected a lower priority than the assigned triage category were significantly less likely to perceive their condition as serious (mean score = 6.1 ± 1.9, *p* ≤ 0.01), urgent (mean = 6.0 ± 2.3, *p* ≤ 0.01), and painful (mean = 5.3 ± 2.6, *p* ≤ 0.01) than the other two categories.

### Multivariate analysis

A multinomial logistic regression analysis was conducted with the variables in Table 3. While these variables were statistically significant in the bivariate analysis, only two variables, Perceived Urgency and Pain Score, reached the final stage (Table 4). Using Nagelkerke test, these two variables explained nearly 12 % of the variance in the dependent variable. However, only Perceived Urgency was significantly associated with expected higher priority (OR: 1.27, 95 % CI: 1.14–1.43).

**Table 4:** Results of multivariate analysis

Factor	Adjusted OR	95 % (CI)	Adjusted OR	95 % (CI)
	Expected higher priority^a^		Expected lower priority^a^	
Perceived urgency	1.273	(1.136–1.426)	0.999	(0.880–1.133)
Pain score	1.069	(0.973–1.174)	0.923	(0.828–1.029)
Pseudo R^2^	Nagelkerke = 0.116			

^a^The reference category is: Concordance. *OR* Odds Ratio, *CI* Confidence Interval

## Discussion

Our study showed little agreement between patient perceived priority and actual nurse assigned triage category. There is a paucity of comparable studies in this area. Studies on inter-rater agreement have pre-dominantly focussed on the validation of the triage scales through the eyes of different health practitioners such as nurses, emergency physicians and general practitioners [26, 29–31].

Our findings are consistent with a recent study of concordance between patients’ and their doctors’ assessment of urgency levels in a Norwegian emergency outpatient clinic [32]. The study showed low agreement between doctors and patients (Kendall tau-b = 0.202, *p* < 0.001). However, while country of birth was not associated with inter-rater agreement in our study, the Norwegian study showed that doctors were significantly more likely to assess African patients with higher priority [32].

A study of 73 non-urgent self-referrals to an ED in Israel compared the patients’ evaluation of the urgency with the treating nurses’ evaluation [33]. The results found statistically significant differences between the two groups. Whilst over 77 % of the patients rated their condition as “urgent to most urgent”, only 21 % of the nurses rated the patients’ condition as urgent to most urgent [33]. The study, however, did not analyse the inter-rater agreement between the patients and nurses.

Earlier studies have shown similar results. A 1980 study in USA showed that 44.4 % of the patients thought they needed care immediately, 28.5 % urgently, and 15.6 % promptly. However, the emergency physicians’ prospective assessments were 12.6 %, 26.3 % and 28.1 %, respectively [34]. Another similar study in 1996 confirmed the discordance between patients’ and physicians’ assessment of the urgency of the needed care, although with different magnitudes [35]. In this latter study, 31.7 % of the patients thought they should be seen immediately, 33.6 % urgently, and 21.2 % promptly, whereas the physicians’ assessments were 14.8 %, 22.8 % and 28.2 %, respectively.

Notably, although the multivariate analysis (Table 4) did not yield statistical significance for most variables (except for Perceived Urgency), the findings and the directions of the associations can have important implications. Our findings clearly confirmed the discrepancy between patient perception of urgency and staff assessment of urgency. Respondents seemed to expect a higher priority if they had attended the ED more than once in the past six months, possibly indicating the presence of significant co-morbidities or experience with the system. They considered their condition to be serious and required urgent care.

Our findings also indicate the complex and delicate nature of the patient–health practitioner relationship, particularly when the patient’s expectation is not met as a result of allocating a lower triage category. While there are some suggestions that patients should be able to have input into their triage assessment, opponents of this view point to issues such as ethics (fairness, justice and equity), resource limitations, actual ability to meet the patients’ expectations, and the level of knowledge, education and clinical decision making that goes into triage assessment that most patients do not have [13, 18, 36, 37]. Furthermore, involving the patients’ input would require the development and implementation of specific patient education programs, which are hampered by cost and lack of evidence of their success.

Although brief, triage is a complex decision making process, performed by experienced and trained ED registered nurses [26]. It is deigned to sort and prioritise care to reduce the negative impact on the prognosis of a prolonged delay before treatment [38]. The triage process itself requires engagement and two way communication with the patient and, where appropriate, their family. However, improvements in this process may be required so that triage can become more patient centred. Further research is needed to investigate the patient’s perceived urgency at the start and end of their ED episode as their perceptions may change as a result of their ED experience, receiving assurance from clinical experts, and seeing other, perhaps sicker, patients in the department.

Of considerable clinical concern is the subgroup of patients (20 %) who tended to under-rate their priority compared to health practitioner assessments. Our study did not show statistically significant factors that may influence this perception. However, there are significant clinical risks in this group that reinforces the need for professional assessment, active and ongoing management of the triage process and professional observation of those waiting. Further research is required to identify the characteristics of those who tend to over or underrate their urgency, and also to understand if there is a correlation between personality type and expectations of either higher or lower triage scores. The significance of pain assessment as a key determinant of the patient urgency perception is important as there are significant personality and cultural influences on not only the perception of pain but also on the public expression of that perception [39].

One key implication of this research relates to the importance of managing around patient expectations. Given that nearly 1 in 2 patients in our study expected to have been allocated a higher priority than they actually were, opportunity exists for health care practitioners to communicate further with patients regarding the triage process which would provide an opportunity for patients to engage in and understand the clinical decision making around their triage category. This addresses the imbalance between expectation and reality through reassurance, by identifying and addressing the patient’s concern. It also allows for a ‘patient voice’ which may incorporate other contextual issues into the assessment that are causing the patient and their carers concern. An example of this ‘patient voice’ is “Ryan’s Rule”, an initiative recently introduced into Queensland Health which allows patients to escalate demand for extra help if they “are concerned about a patient in hospital who is getting worse or not improving” [40]. Given that the triage process is usually fast and short, allowing time for further patient/carer input can improve the safety and quality of care.

### Strengths and limitations

This is one of the first studies to explore in some depth the patient perceptions of their priority and the relationship between that perception and the triage category assigned by triage nurses. It explores the value of the “patient voice” and identifies options for incorporating processes that may better understand and capture those perceptions into the priorities assigned.

The major limitation of this study is its sample size and representativeness. Overall, 697 (43 %) of the 1608 patients attending the EDs did not participate in this study. Since we were unable to access their medical records without their consent, we cannot assess the impact/bias their exclusion from the study might have had on the results. It is possible that there would be a higher concordance between actual triage and patients’ perceptions for those who were seriously ill. Furthermore, only a subgroup of the participants consented for access to their medical records. However, as our analysis showed there were no statistically significant differences between the consented and non-consented participants in major socio-demographic and health related variables. It is also recognised that this study was undertaken in a single state and despite all the efforts to capture a representative sample of the presenting patients, the final sample did not include seriously ill patients for practical reasons. Furthermore, the data collection was conducted in autumn and this may mask seasonal variations in attendances. Further investigations in other locations, times and with different patient groups may be required to enable comparisons and generalisability of the findings.

We asked the respondents to rate their perception of the expected priority before the start of the treatment. However, as some patients had already started their treatment, their retrospective responses may have been affected and biased. Furthermore, our study did not include some other factors that may contribute to differences in patient perceptions such as personality traits, experience, and health literacy. Further research would be of benefit to explore the impact of these and other factors such as geography (e.g. urban vs rural), diagnosis and health system differences (e.g. triage practices in different EDs), particularly for those who tend to overrate or underrate their assessment. This greater understanding may assist with aligning triage process management with patient expectations and thus help achieve improved clinical and organisational satisfaction.

## Conclusions

Our findings show a gap in patient–practitioner understanding of the priority of patients attending emergency departments, which can have implications for the management of the emergency care system. While patient centred care is acknowledged as an important part of system development, improved and open communication and the incorporation of the ‘patient voice’ into the triage process require a careful balance between understanding the patient’s perspectives and the objective assessment of a trained health professional who needs to prioritise the allocation of the resources in accordance with the urgency of the presented conditions. Patients’ involvement in the decision making process and particularly in the triage assessment is sensitive, difficult, and the subject of opposing opinions about its fairness. Further work is required on how to manage the expectations by acknowledging the discrepancy between the two parties.

## Abbreviations

95 % CI: 95 % Confidence Interval
ED: Emergency department
EDIS: Emergency Department Information System
GP: General practitioner
SPSS: Statistical Package for the Social Sciences
